# X-linked lymphoproliferative disease type 1: a clinical and genetic update

**DOI:** 10.3389/fimmu.2025.1620327

**Published:** 2025-06-12

**Authors:** Jiaxun Li, Luyu Lv, Qingqing Wei, Wenpeng Pang, Chunjuan He, Hao Wu, Liping Guo

**Affiliations:** ^1^ Department of Microbiology, School of Basic Medicine, Guangxi Medical University, Nanning, Guangxi, China; ^2^ Guangxi Key Laboratory of Thalassemia Research, Guangxi Medical University, Nanning, Guangxi, China; ^3^ Department of Clinical Laboratory, First People's Hospital of NingYang, Taian, Shandong, China; ^4^ Key Laboratory of Basic Research Regional Diseases (Guangxi Medical University), Education Department of Guangxi Zhuang Autonomous Region, Nanning, Guangxi, China

**Keywords:** X-linked lymphoproliferative disease 1, SH2D1A gene, Epstein-Barr virus, SAP (signaling lymphocyte activation molecule-associated protein), hematopoietic stem cell transplantation, treatment

## Abstract

X-linked lymphoproliferative disease (XLP), also known as Duncan’s disease, is a primary immunodeficiency disorder linked to the X chromosome. In 1998, SH2D1A, which encodes the signaling lymphocyte activation molecule (SLAM)-associated protein (SAP), was identified as the first pathogenic gene associated with XLP. To date, more than 100 mutation sites in this gene have been documented. The disease is associated with infection with Epstein-Barr virus (EBV) and characterized by hemophagocytic lymphohistiocytosis (HLH), hypogammaglobulinemia, and lymphomas. Pathogenesis is intricately associated with cell type-specific SAP-SLAM signaling pathways. Particularly, the immune cell defects involve impaired T cell-B cell interactions, reduced cytotoxicity of Natural Killer (NK) cells, and abnormal development of Natural Killer T (NKT) cells. These factors collectively increase susceptibility to EBV and drive clinical manifestations in XLP type 1 (XLP1) patients. Although establishing a definitive correlation between specific genotypes and clinical phenotypes remains challenging, emerging evidence suggests a potential association. This underscores the critical need for further large-scale studies to elucidate this relationship. Given the current understanding of the pathophysiological mechanisms associated with XLP1, specific treatments to normalize SAP expression and restore immune tolerance in XLP1 patients play an important role. In addition to the necessity for long-term studies to verify the efficacy and safety of hematopoietic stem cell transplantation (HSCT), gene therapies currently under development, along with other emerging treatments, exhibit substantial promise for future clinical applications.

## Introduction

X-linked lymphoproliferative disease (XLP) was first described in the 1970s by Purtilo et al, who identified a familial predisposition to fatal outcomes following Epstein-Barr virus (EBV) infection, characterized by fulminant hepatitis, lymphoproliferation, and immunodeficiency (1). In the 1980s, the establishment of the XLP Registry revealed a mortality rate of ~75%, primarily due to hepatic necrosis and multi-organ failure post-EBV exposure (2). Subsequent genetic studies delineated two distinct subtypes: XLP type 1 (XLP1), caused by mutations in SH2D1A (encoding signaling lymphocytic activation molecule [SLAM]-associated protein [SAP]), and XLP type 2 (XLP2), linked to BIRC4 (encoding X-linked inhibitor of apoptosis [XIAP]) defects. While XLP2 is more closely associated with EBV-driven clinical features such as hemophagocytic lymphohistiocytosis (HLH), splenomegaly and colitis (3, 4). This review focuses on XLP1, where SH2D1A mutations impair immune cell signaling, predisposing patients to EBV-driven pathologies even before classical symptom onset.

XLP1 is characterized by defective EBV clearance. EBV, a lymphocryptovirus of the γ-herpesvirus family, transforms B cells through latent growth programs. EBV infects over 95% of the world’s population (5). In immunocompetent individuals primary EBV infection is usually asymptomatic, but in immunocompromised patients, it can result in severe disease such as infectious mononucleosis (IM) associated with polyclonal B cells expansions, abnormal proliferation of B cells may even lead to lymphomas.

Due to the rarity and complexity of genetic and clinical phenotypes of XLP1, as well as the incomplete understanding of its pathogenic mechanisms, patients with XLP1 often fail to receive timely and effective treatment. However, advancements in gene editing, targeted therapies, and immune reconstruction technologies have brought new hope for the treatment of XLP1. This review aims to provide the comprehensive insights into XLP1, focusing on its clinical and genetic characteristics, molecular mechanisms, diagnosis methods and the latest treatment strategies.

## Genetic landscape of XLP1

**Figure 1:**
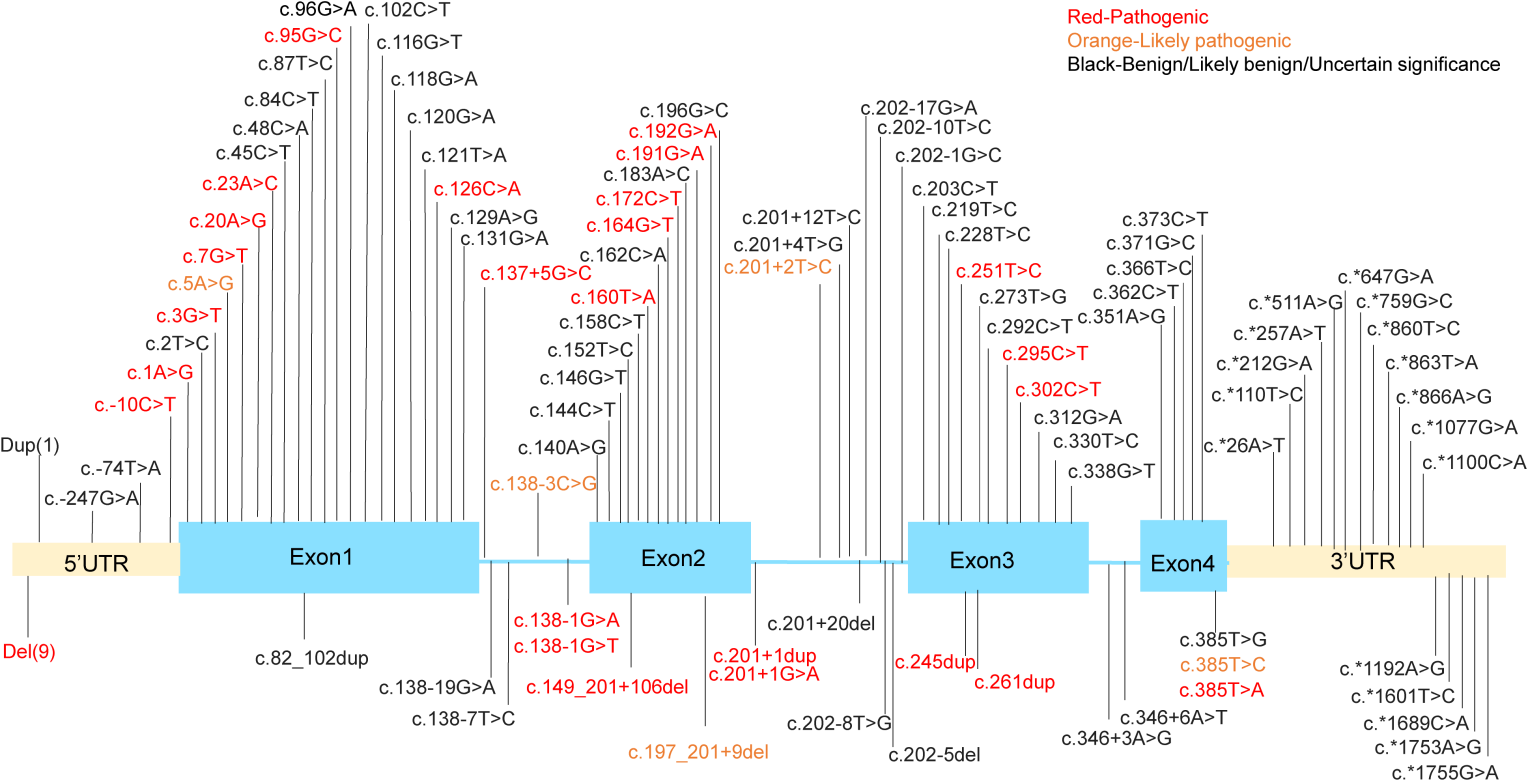
The mutation sites in the SH2D1A gene. Dup (1) means g. (? 123480147)_(123505241_)? dup. Del(9) means g.(?_123480147)_(123480649_)?del, g.(? 123494030)_(123499653_)?del, g.(?_123497345)_(123505232_)?del, g.(?_123499591)_(123499694_)?del, g.(?_123499591)_(123505241_)?del, g.(? 123499639)_(123506985_)?del, g.(?_123504006)_(123505241_)?del, g.(?_124346297)_(124371411_)?del, g.(?_124346562)_(124346780_124365760)del.

SH2D1A gene is located in the q25 region of the X chromosome, spanning 40kb of genomic sequence and containing four exons. It encodes SAP, a protein that functions as a molecular switch, enabling SLAM family members to act as either activating or inhibitory receptors (6, 7). This protein is almost entirely composed of a single Src Homology 2 (SH2) domain (8, 9). XLP1 has been reported globally (10, 11), and is shown to be associated with a deficiency of SH2D1A gene. The mutations in SH2D1A gene associated with XLP1 manifest in various forms, including deletions, nonsense mutations, missense mutations, and splicing abnormalities. In recent years, with the increasing number of XLP1 cases, more rare and complex mutation forms have been identified (12–15). We performed a comprehensive analysis of known genetic mutations in SH2D1A based on the ClinVar database of the National Center for Biotechnology Information (https://www.ncbi.nlm.nih.gov/clinvar/?term=%22SH2D1A%22%5BGENE%5D&redir=gene) (**Figure 1**). Among nearly 150 variants cataloged in ClinVar to date, approximately one-third are classified as pathogenic or likely pathogenic.

Among the mutations identified, truncating mutations account for approximately 40% of reported cases and generally lead to a complete loss of SAP expression. For example, the recurrent nonsense mutation c.163C>T (p.Arg55*) introduces a premature stop codon, thereby eliminating the SH2 domain that is critical for SAP’s interaction with SLAM receptor. Similarly, large deletions spanning exons 2–4 (e.g., 124,506_127,891del) remove the entire coding sequence, leaving patients without functional SAP (14). Missense mutations are also particularly significant as they frequently occur in highly conserved regions of exons and constitute about one-third (33.00%, 34/103) of variants. These mutations predominantly cluster within the SH2 domain (residues 7-102), which mediates phosphotyrosine binding. The p.Gly49Val substitution, located in a highly conserved β-sheet region, disrupts SAP’s ability to recruit Fyn kinase to SLAM receptors. Surface plasmon resonance (SPR) assays reveal a 100-fold reduction in binding affinity (Kd = 1.5 μM compared to 15 nM for wild-type SAP), impairing downstream phosphorylation of Vav1 and cytoskeletal reorganization in T cells (16). Other notable missense variants, such as p.Arg32Trp and p.Thr68Ile, destabilize the tertiary structure of the SH2 domain, as confirmed by nuclear magnetic resonance (NMR) spectroscopy (9).

## Clinical manifestations

The main clinical features of XLP1 include HLH, hypogammaglobulinemia, and lymphoma. Among these, HLH is the most frequent and life-threatening presentation, occurring in 35.2% of XLP1 patients and typically triggered by primary EBV infection (4). Most cases are reported in individuals of East Asian descent, suggesting a potential geographic distribution pattern in the pathogenesis of EBV-associated HLH (17). Recent studies have identified impaired responsive proliferation of CD8 + T cells and upregulation of type I interferon (IFN) signaling as key characteristics commonly observed in EBV-HLH cases (18). HLH presents with fever, hepatosplenomegaly, cytopenia, rash, lymphadenopathy, jaundice, and edema, affecting 60-75% of XLP1 patients and leading to significant nervous system damage and high mortality (19). Patients may exhibit any combination of these clinical criteria; atypical presentations are not uncommon, and conditions mimicking HLH can complicate clinical management (20). Notably, recent research indicates that despite a reduced mortality rate to about 65.6%, with rapid progression to death within two months in some EBV-induced HLH cases (21). It is important to highlight that approximately 21% of HLH cases occur independently of EBV infection, with the underlying trigger remaining unknown (22).

It should be noted that EBV infection in individuals without XLP can present as the well-recognized condition IM, which is a severe clinical manifestation associated with mortality rates exceeding 90% in such cases (23). IM most commonly results from a primary EBV infection. While most symptoms of EBV-associated IM resolve within a month, however, 10-13% of young adults experience prolonged recovery periods lasting up to 6 months after the initial onset of IM symptoms, characterized by persistent pharyngitis and fever (24). However, previous studies have failed to identify specific clinical features or laboratory parameters that reliably predict protracted recovery in IM. Recently, a study found that the percentage of CD8^+^ atypical mononuclear cells could potentially serve as a promising biomarker for predicting the anti-EBV immune response in IM patients (25).

Up to 50.5% of XLP1 patients exhibit a spectrum of humoral immune abnormalities, frequently characterized by reduced levels of one or more immunoglobulin subclasses, most notably decreased IgG serum concentrations, alterations in IgM and/or IgA serum concentrations, and occasionally abnormally elevated values. Such immunological changes may predispose individuals to recurrent infections, particularly respiratory tract infections (26, 27).

Nearly 30% of patients develop lymphoproliferative disease (lymphoma), specifically high-grade B-cell non-Hodgkin type lymphomas. These lymphomas in XLP1 are predominantly extranodal, with approximately 75% of lymphomas occurring in the ileocecal region. Notably, lymphomas can develop during childhood and may even arise before EBV exposure. While remission can be achieved through chemotherapy, relapse, the development of a second lymphoma, or other manifestations of XLP1 are frequently observed (26, 28).

### Atypical presentations

In addition to common symptoms, recent reports have highlighted rare clinical manifestations in XLP1 cases, such as aplastic anemia, lymphocytic vasculitis, chronic gastritis, skin lesions, hemorrhagic enteritis, and EBV-related lymphohistiocytic infiltration of the eye orbits and sinuses, which showed responsiveness to rituximab treatment (29–32). While most XLP1 patients experience onset before age 10, adult-onset cases are also clinically significant. Cases include but not limited to a 31-year-old with EBV-HLH and T-cells lymphoma, a 20-year-old with recurrent fever and bilateral pneumonia, and a 49-year-old developing multi-infarct dementia (33–35). Besides, female XLP1 patients are often overlooked; Liang et al. reported a 44-year-old female who developed acute-onset EBV-HLH followed by rapidly progressing natural killer (NK) cell leukemia (36), highlighting the complexity of the clinical phenotype. Collectively, these cases demonstrate that XLP1 can lead to acute or chronic damage affecting multiple organs and systems in an unpredictable and irregular manner.

## Genotype-phenotype discordance

Despite significant advances in genetic profiling, predicting clinical outcomes based on SH2D1A mutations remains a formidable challenge. Strikingly, identical germline mutations can manifest as divergent phenotypes within the same family (21, 37). This variability highlights the potential roles of epigenetic modifiers and environmental factors in shaping disease manifestation. It has been hypothesized that missense and nonsense mutations at specific loci may restore partial of CD8^+^ T cell function, resulting in milder clinical phenotypes in affected individuals (38). Early studies indicated that patients with missense mutations in SH2D1A tends to exhibit milder clinical presentations compared to those with truncating mutations that produce nonfunctional SAP proteins (39). Additionally, while not all nontruncating missense mutations in the SH2 domain have been clinically correlated, one previously reported case demonstrated that substitution of an adjacent AA (49 Gly→Val) was associated with the development of EBV-associated Burkitt lymphoma (11). Therefore, the observed clinical heterogeneity between genotype-phenotype of XLP1 should be interpreted with caution. As research continues, further insights into the molecular mechanisms underlying the progression of specific certain mutation forms to distinct clinical manifestations may emerge.

In addition, although XLP1 patients are highly susceptible to EBV, approximately 35% of them have no documented history of prior EBV infection (40). Among EBV-positive patients, the incidence of HLH is significantly higher (51% in EBV-positive patients compared to 21.4% in EBV-negative patients), while the rates of dysgammaglobulinemia (37.2% in EBV-positive patients versus 52% in EBV-negative patients) and lymphoma (19.6% in EBV-positive patients versus 25% in EBV-negative patients) are relatively lower. Furthermore, there is no significant difference in mortality when compared to EBV-negative patients (approximately 30%) (21, 27). Therefore, XLP1 should be considered an immune dysregulation disorder that is not exclusively triggered by EBV infection. The association between EBV infection and specific genotypic mutations remains unclear, necessitating future research to clarify clinical distinctions between EBV-associated and non-EBV-associated XLP1 and their genotypic correlations.

## Immunological features and pathogenesis

**Figure 2:**
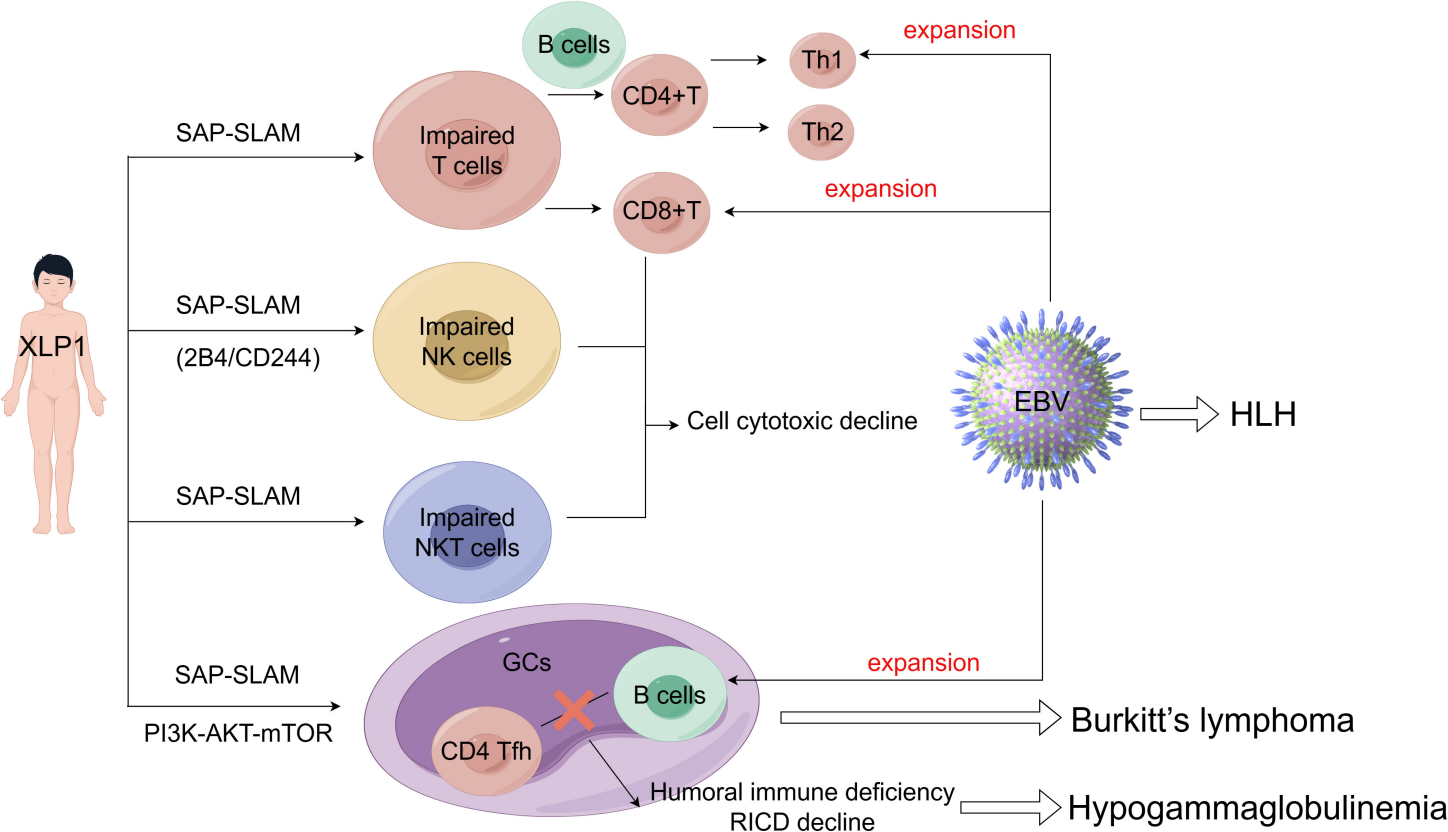
The diagnostic flow chart for XLP1.

The pathogenesis of XLP1 is intricately associated with the cell type-specific expression of SLAM-SAP. SAP is predominantly expressed in T cells, NK cells, and Natural Killer T (NKT) cells, where it modulates SLAM family receptor (SFR)-mediated signaling pathways, but is minimally expressed in B cells (41). In XLP1, loss-of-function mutations in SH2D1A disrupt critical immunoregulatory circuits within these lymphocyte populations. For instance, SAP deficiency in CD8^+^ T cells and NK cells impairs cytotoxicity against EBV-infected B cells, while defective SAP-dependent T follicular helper (TFH) cell function compromises germinal centers (GCs) formation and humoral immunity. Conversely, despite low SAP expression in B cells, their dysregulation in XLP1 may arise secondarily from aberrant T cell-B cell interactions or unchecked EBV-driven proliferation. Collectively, these cell-specific functional deficits manifest as the clinical hallmarks of XLP1, including HLH, hypogammaglobulinemia, and lymphoma (**Figure 2**). The critical role of SAP in coordinating immune cell crosstalk and effector functions underscores its centrality in maintaining immune homeostasis and elucidates the multisystemic pathology observed in XLP1.

### T/B cells

SAP plays an important role in the normal immune function of T cells, including the CD4^+^ T cells, CD8^+^ T cells, and T helper (Th) cells. In the absence of SAP expression, tyrosine residues on SLAM family members recruit several potent inhibitory molecules, such as SH2 containing protein tyrosine phosphatase (SHP)-1, SHP-2 and the SH2 containing inositol 5’-phosphatase proteins (SHIP) (42). These inhibitory molecules induce T cells inhibitory signals, thereby blocking the activation, development, and immune function, leading to abnormal T cell immune responses and reduced cytotoxicity (43). Unlike the unstable interactions between B cells interaction, SAP-deficient T cells, SAP-deficient T cells can still form stable conjugates with dendritic cells, suggesting that impaired SLAM-SAP signaling primarily affects T-B cell interactions. Indeed, SAP-deficient T cells are initially activated normally by antigen-presenting dendritic cells in response to immunization and infection. However, they fail to differentiate into mature CD4^+^ follicular helper T cells (CD4 Tfh), a process that requires cooperation with normal B cells. Specifically, SAP deficiency severely impairs the adhesion of CD4 Tfh cells to B cells in GCs, resulting in compromised humoral immunity and hypogammaglobulinemia. It appears that the gain of SAP-independent SFRs inhibitory signaling suppresses the entry of Tfh cells into GCs (44–46).

Furthermore, impaired T cell development exacerbates B cell-mediated humoral immune deficiency and diminishes sensitivity to restimulation-induced cell death (RICD), thereby resulting in an ineffective immune response against pathogen infection (21). RICD is an apoptotic program that regulates effector T cell expansion, triggered by repeated stimulation through the T cell receptor (TCR) in the presence of interleukin-2 (IL-2) (47). CD4^+^ regulatory T cells (Tregs) consume IL-2 and undergo frequent TCR stimulation;consequently, they exhibit high resistance to RICD. T cells from XLP1 patients lack the adaptor molecule SAP, also display resistance to RICD (48, 49). A recent study revealed that normal Tregs express very low levels of SAP compared to conventional T cells. Forkhead box P3 (FOXP3) reduces SAP expression by directly binding to and repressing the SAP promoter. These findings elucidate the mechanism behind FOXP3-mediated RICD resistance in Tregs (49). Furthermore, T cells from patients with XLP1, who lack functional SAP, exhibit hyper-responsive to PD-1 signaling. Conversely, overexpression of SAP abrogated the inhibitory effect of PD-1. Thus, while RICD is impaired in SAP deficiency, PD-1 signaling is enhanced, potentially as a compensatory mechanism to maintain T cell signal regulation and balance. In summary, SAP governs several critical signaling pathways in both T cells and B cells. Impaired SLAM-SAP signaling affects T cell-B cell interactions, the development of TH2 and Tfh cell subsets, as well as GCs formation (50, 51).

Since a hallmark feature of XLP1 is its high sensitivity to EBV infection, Palendria et al. developed an innovative XLP1 vector model to elucidate the underlying immune deficiency characteristics behind this specific vulnerability. Their findings revealed that SAP deficiency in T cells impairs interactions with B cells while leaving interactions with other antigen-presenting cells unaffected. This specific defect not only clarifies the molecular pathogenesis of the increased susceptibility to EBV infection in XLP1 patients but also sheds light on their elevated risk of developing B-cell lymphomas (52). Interestingly, EBV is the only known human pathogen that selectively infects B cells, and two different hypotheses have been proposed to describe this process. The first suggests that EBV directly infects memory B cells. The second posits that EBV initially infects resting naïve B cells, which subsequently enter GCs, where viral genes are moderately expressed. GCs serve as the site of antibody class switching of B cells in response to pathogens (46, 53). As noted earlier, SAP deficiency severely compromises the adhesion of CD4^+^ Tfh cells to B cells in GCs, leading to the upregulation of SLAM family ligands necessary for effective T-B cell cross-talk and immune function.

After EBV infection, the body’s antiviral immune response is primarily mediated by the production of IgM antibodies against EBV viral capsid antigen and a substantia expansion of CD8^+^ T lymphocytes targeting EBV-infected B cells (54). In the context of SAP deficiency, T cells can downregulate Th2 response; however, EBV infections can upregulate Th1 response, leading to an imbalance.between Th1/Th2. This Th1/Th2 imbalance is a well-documented mechanism of immune escape. Consequently, the defect may contribute to the high recurrence rate of EBV infection in XLP1 patients (55–58).

### NK/NKT cells

In addition to T cells and B cells, initial functional defects observed in lymphocytes from XLP1 patients included impaired NK cells activity (59). Subsequent studies demonstrated that SAP couples the SFRs to Fyn (a Src family protein tyrosine kinase) via arginine 78 (R78) in the SH2 domain, triggers Vav-1(an exchange factor promoting cytoskeleton reorganization and lytic synapses formation) phosphorylation thereby enhancing NK cell conjugate formation (60, 61). Chen et al. revealed that SFRs are entirely inhibitory in SAP-deficient NK cells, thus, removing SFRs from SAP-deficient NK cells completely abolishes the impaired NK cells cytotoxicity (62). SFRs can transmit two distinct signaling pathways: SAP-dependent and SAP-independent. These two pathways play opposing roles at least in NK cell activation. SAP-independent SFR signaling appears to promote NK cell functional competence, whereas SFRs as self-specific activation receptors can desensitize NK cell responsiveness (63). In the absence of SAP, inhibitory molecules, such as SHIP-1, associate with SFRs to suppress NK cell activation, a phenomenon observed in NK cells from XLP1 patients (64, 65).

Members of the SLAM family, including 2B4/CD244, CD352, and CD319, have been identified as cytotoxic receptors expressed on NK cells (16). Among these, 2B4 plays a critical role in immune regulation by coactivating NK cell cytotoxicity and cytokine secretion. The immune-modulatory function of 2B4 is dependent on the small intracellular signaling molecule SAP (66). Studies have demonstrated that the ability of 2B4 to enhance NK cells cytotoxicity is abolished in SAP-deficient NK cells (67).

The functional impairment of NK cells is further exacerbated by the educational process during NK-cell maturation, which is intricately linked to the inhibitory 2B4/CD48 pathway. SAP-deficient NK cells, which lack self-HLA class I specific inhibitory NK receptors (self-iNKRs), display autoreactivity toward CD48-negative targets, including mature dendritic cells (DCs). This aberrant cytotoxicity impairs antigen presentation and disrupts adaptive immunity, thereby perpetuating a vicious cycle of immune dysregulation (62, 68, 69). Although EAT-2, a SAP-related adaptor protein, can partially compensate for the loss of SAP by inducing calcium flux and Erk activation and preventing SFR binding with SH2 domain-containing inhibitory molecules through Y127-dependent recruitment of PLCγ, it is insufficient to fully restore normal NK cell function in the absence of SAP (64).

In particular, NK cells constitute the primary lymphocyte subset of the innate immune system responsible for mediating antiviral responses. It has been demonstrated that specific subsets of NK cells play a critical role in the early control of EBV infection (70, 71). Earlier research revealed that the impaired function of NK-T-B-antigen (NTB-A, CD352) is pivotal in the failure of XLP-NK cells to eliminate EBV-infected target cells (72). Subsequent studies further elucidated that engagement of the 2B4 receptor selectively inhibits immunoreceptor tyrosine-based activation motif (ITAM)-dependent activating receptors,including natural cytotoxicity receptor (NCR) and CD16, while sparing NK group 2 member D (NKG2D) and DNAX accessory molecule-1 (DNAM-1) (73). This selective inhibition specifically impairs the clearance of EBV-infected B cells, thereby contributing to the increased susceptibility to EBV infection the increased in XLP1 patients (74).

NKT cell development is critically dependent on SAP-SFR signaling, as evidenced by the specificity of SAP with SFRs (75). In both SAP-deficient mice and humans, a severe depletion of NKT cell occurs due to a developmental block at early stages, which compromises immune surveillance against EBV and/or B cells lymphoma in XLP1 patients (7, 62, 76, 77). While SAP-dependent SFR signaling is essential for NKT cell development, SAP-independent SFR signaling plays only a minor role in this process. However, SAP-independent SFR signaling can completely impair NK cell function and TFH cell-mediated humoral immunity (62, 78). The combined loss of NKT cells and dysfunctional NK cells establishes a “two-hit” model of immune failure in XLP1: impaired innate cytotoxicity leads to unchecked EBV, while compromised tumor surveillance synergizes to drive HLH and lymphoma.

### Signal transduction pathway

Emerging evidence highlights aberrant activation of the phosphatidylinositol-3-kinase (PI3K)-AKT-mTOR signaling pathway as a critical pathogenic mechanism in XLP1. Wang et al. demonstrated constitutive hyperactivation of this pathway in XLP1 patients, which contrasting sharply with its partial or absent activation in healthy individuals or those with HLH of other etiologies (79). Given the well-established role of PI3K signaling in lymphocyte development, differentiation, and GCs formation, dysregulation of this pathway may underlie the humoral immune defects observed in XLP1. For example, PI3K-deficient murine models exhibit impaired GC reactions, reduced proliferation of mature B cells, and severe humoral dysfunction (80, 81). Although these findings suggest a connection between PI3K hyperactivity and clinical manifestations of XLP1, the precise regulatory interplay between SAP and the PI3K pathway remains unclear. Key unresolved questions include whether SAP directly modulates PI3K activity, whether its loss triggers compensatory signaling cascades, and what molecular mechanisms drive of pathway hyperactivation in the absence of SAP.

Beyond humoral defects, SAP deficiency renders XLP1 patients susceptible to rare autoimmune complications. In XLP1-associated limbic encephalitis, anti-α-amino-3-hydroxy-5-methyl-4-isoxazolepropionic acid receptor (AMPAR) autoantibodies generated by dysregulated B cells have been implicated in central nervous system (CNS) pathology (82). Nevertheless, the mechanisms driving other neurological manifestations, such as cerebral vasculitis, or hematological abnormalities like aplastic anemia, remain incompletely understood. These knowledge gaps underscore the necessity of investigating how SAP deficiency broadly disrupts immune tolerance across multiple systems.

The pathogenesis of EBV-driven complications in XLP1, particularly HLH, involves intricate interactions between viral tropism and dysregulated immune responses. EBV preferentially infects CD5dim HLA-DR^+^ CD8^+^ T cells, leading to oligoclonal or monoclonal expansion of infected lymphocytes, with NK cell infection documented in approximately 20% of cases (83). Recent studies have highlighted impaired CD8^+^ T-cell proliferative responses and enhanced type I IFN signaling as key features of EBV-HLH (18). Interestingly, the mechanisms underlying HLH development in EBV-negative XLP1 patients remain largely elusive, suggesting SAP-independent pathways of immune hyperactivation. Furthermore, while EBV infection is known to promote B-cell transformation and increase lymphoma risk, paradoxically lower lymphoma incidence has been reported in EBV-positive XLP1 patients compared to their EBV-negative counterparts. This observation points to undefined immune surveillance mechanisms that differentially regulate EBV-associated versus spontaneous lymphomagenesis in the context of SAP deficiency.

Critical unanswered questions remain regarding the clinical heterogeneity of XLP1. First, the relationship between residual SAP expression levels and susceptibility to EBV infection or disease severity has yet to be fully characterized. Second, it remains unclear whether SAP deficiency directly enhances EBV tropism for specific lymphocyte subsets or simply allows uncontrolled viral proliferation due to cytotoxic defects. Addressing these issues will necessitate longitudinal studies that correlate SAP expression levels, immune cell functionality, and clinical outcomes in both EBV-positive and EBV-negative cohorts.

## Examination and diagnosis methods

The diagnosis of XLP1 necessitates a comprehensive and systematic approach that integrates clinical suspicion, laboratory testing, and genetic validation. HLH is diagnosed according to the HLH-2004 criteria (≥5/8 clinical/laboratory features) (84). However, distinguishing between primary/genetic HLH and secondary HLH is critical, especially in patients with acute or fulminant EBV infection, EBV-associated HLH, or HLH triggered by other viruses such as cytomegalovirus or adenovirus in pediatric and adolescent populations (85). EBV detection remains pivotal in evaluation; first-line tests include PCR for EBV-DNA load, IgM antibodies, and heterophile antibodies. Advanced techniques, such as PrimeFlow™ RNA assay (with a sensitivity of detecting 0.01% EBV-infected cells) and EBER flow fluorescence *in situ* hybridization (FISH), further enhance diagnostic sensitivity and provide deeper mechanistic insights into EBV-associated pathologies (86, 87). Importantly XLP1 should also be considered in cases of EBV-negative HLH.

Immunological profiling plays a critical role in diagnosis, particularly for conditions such as hypogammaglobulinemia (see Clinical Features) and reduced peripheral NKT cell counts (though normal counts do not rule out XLP1) (77, 88). A higher level of suspicion is warranted in patients with B-cell lymphoproliferative disorders, especially those who develop secondary distinct lymphomas following initial remission from non-Hodgkin lymphoma treatment (28). Functional assessment of SAP protein expression using flow cytometry (FCM) serves as an effective rapid screening tool; however, in cases with a clinically compatible presentation, secondary validation of the inhibitory effect of the 2B4 receptor is necessary to confirm the findings. Combining SAP expression analysis in peripheral blood NK cells with a functional assay of the 2B4 receptor enables accurate identification of XLP1 (31, 89).

Genetic testing serves as the diagnostic gold standard. For patients with a high clinical suspicion or family history, sanger sequencing of SH2D1A is recommended as the initial approach. If results are inconclusive, advanced techniques such as amplicon sequencing, next-generation sequencing (NGS), whole-exome sequencing (WES), or whole-genome sequencing, should be pursued (90, 91). Emerging techniques, like droplet digital PCR (ddPCR), provide cost-effective and sensitive mutation detection for small genes or familial studies, though further optimization is required for broader application (92).

**Figure 3:**
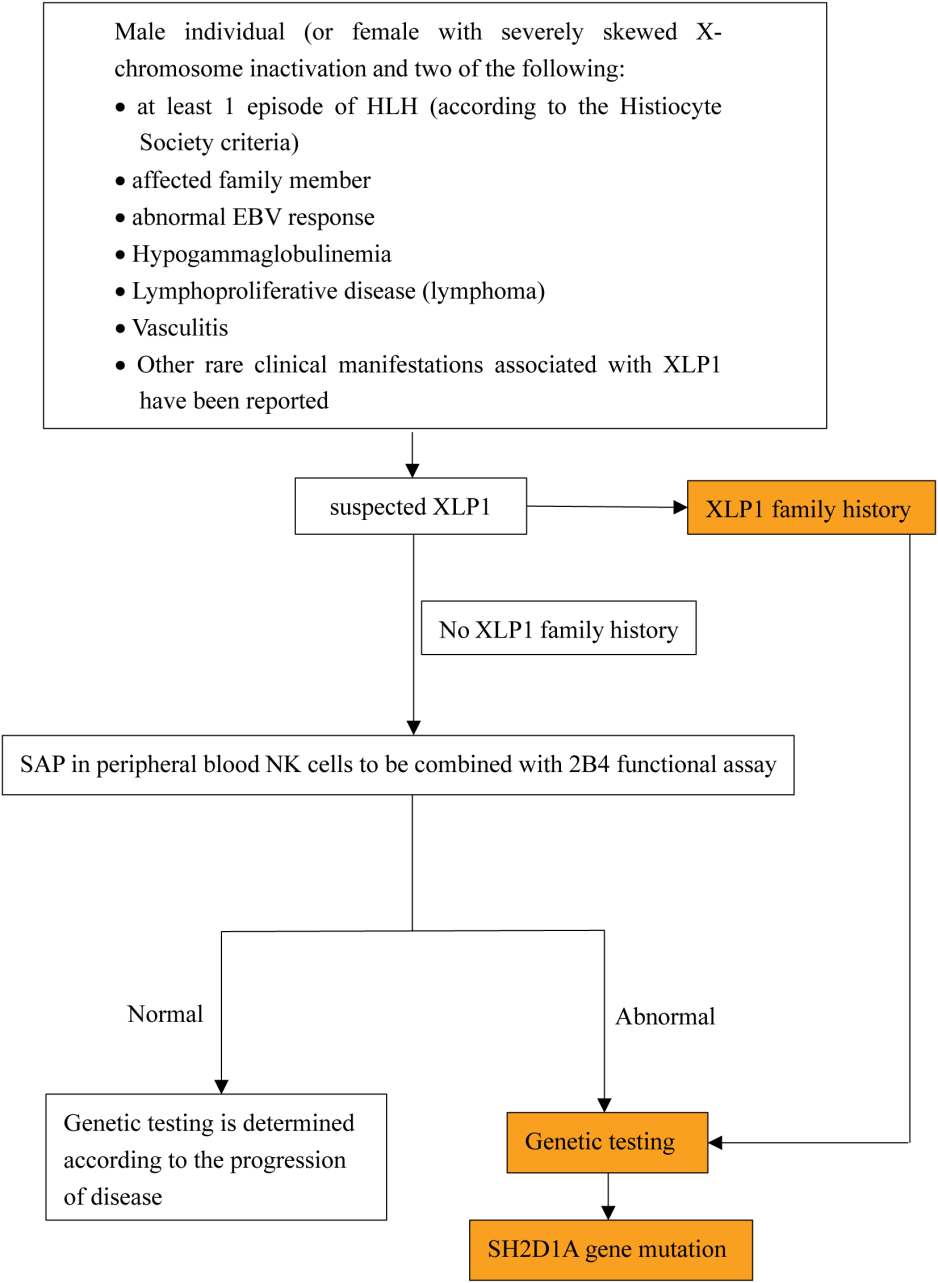
The pathogenesis and clinical features of XLP1.XLP1 is tightly linked to the cell type-specific SAP-SLAM signaling (may also include PI3K-AKT-mTOR signal pathway). Particularly the immune cell defects including impaired T cell-B cell interactions, NK cells cytotoxicity and NKT cell development. These combined factors contribute to an increased susceptibility to EBV and other clinical manifestations seen in XLP1 patients, including HLH, hypogammaglobulinemia, lymphoproliferation and lymphoma.

Diagnostic challenges remain significant due to overlapping features with XLP2, asymptomatic cases without a family history, and clinically overlooked EBV-negative presentations. We outline a diagnostic approach based on the current XLP1 diagnostic criteria established by the European Society for Immunodeficiencies (ESID) 2019 guidelines (https://esid.org/Working-Parties/Registry-Working-Party/Diagnosis-criteria/) (summarized in **Figure 3**). This approach highlights the importance of recognizing early clinical warning signs, such as abnormal EBV response, HLH, hypogammaglobulinemia, and lymphoma, which are ultimately through genetic testing. The future development of cost-effective genetic screening methods or biomarkers with high sensitivity and specificity may enhance early diagnosis and reduce mortality associated with delayed interventions.

## Current treatment and potential therapies for XLP1

### Symptomatic supportive treatment

The management of XLP1 focuses on symptom control and complication prevention. Continuous monitoring of EBV viral load is essential to reduce the risk of recurrent infections and life-threatening complications, such as HLH. Rituximab, an anti-CD20 monoclonal antibody, effectively decreases EBV viremia by depleting infected B cells. However, it also poses the risk of B-cell depletion and prolonged hypogammaglobulinemia (93). Additionally, in some cases of XLP1, T cells may also be infected. If high EBV-DNA copy numbers persist after rituximab administration, it is important to consider the possibility of infection in cell types other than B cells (87). Immunoglobulin replacement therapy is used to manage hypogammaglobulinemia-related infections, while B-cell lymphomas are treated with standard chemotherapy followed by expedited HSCT.

### HLH-specific therapies

HLH treatment adheres to the HLH-1994 protocol (comprising etoposide, dexamethasone, and delayed cyclosporine ± intrathecal (IT) methotrexate), achieving a 5-year survival rate of 54% despite risks associated with early mortality, transplant-related complications and neurologic late effects (94). the HLH-2004 protocol introduced upfront cyclosporine and included IT corticosteroids. While HLH-2004 achieved an estimated 5-year survival rate of 62% and reduced pre-HSCT mortality from 27% to 19% compared with HLH-1994, these modifications did not result in a significant improvement in the overall outcome. Therefore, the HLH-1994 regimen remains the current standard of care in most centers (84, 94, 95). The guidance for use of the HLH-1994 protocol issued by HLH Steering Committee of the Histiocyte Society provide us a framework for guiding treatment decisions in this severe disease (96).

Some alternative therapeutic approaches for HLH have been initiated to enhance pre-HSCT survival. Emapalumab is the first cytokine-targeting therapy approved specifically for treating HLH, marking a shift away from cytotoxic chemotherapy, toward more targeted immune modulation (97). Other potential approaches include Antithymocyte globulin (ATG) in combination with etoposide (NCT01104025), alemtuzumab (NCT02472054), tocilizumab (NCT02007239), ruxolitinib (NCT02400463), and a targeted anti-IFN gamma monoclonal antibody (NCT01818492) (84). Notably, ruxolitinib was first reported as a treatment for pediatric HLH in 2017 (98). Two recent studies demonstrated that ruxolitinib could be effective for the initial treatment of secondary HLH both in adults and pediatric patients, showing favorable responses associated with reduced levels of serum cytokines in most cases (99, 100). Several additional studies further described favorable outcomes when ruxolitinib was used as a part of salvage therapy or a bridge to HSCT (101–105). These alternative therapeutic approaches may improve patient survival during the transition to HSCT; however their efficacy and safety profiles should be validated in larger, more diverse patient populations.

### Hematopoietic stem cell transplantation

HSCT remains the only curative option for XLP1, with an 81.4% survival rate in transplanted patients compared to 62.5% in non-transplanted cohorts. Among those non-transplant patients who developed HLH, the survival rate was only 18.8% (21). Another cohort study conducted in Japan involving 33 patients with XLP1 found that 21 of the patients (65%) who did not undergo a transplant died of the disease and complications, 12 patients underwent HSCT, and 11 of these (92%) survived, indicating that HSCT can significantly affect the prognosis and outcomes of XLP1 patients (31). Notably, HLA haploidentical HSCT (haplo-HSCT) from family members using Beijing Protocol is a promising approach due to its cost effectiveness and favorable outcomes (106). Optimal outcomes require HLA-matched donors to minimize graft-versus-host disease (GvHD). A reduced toxicity busulfan-fludarabine conditioning regimen provides low toxicity, a low incidence of GvHD, durable myeloid engraftment, and excellent survival rates, making it potentially suitable for various primary immune deficiencies (107).

However, even with a suitable donor, XLP1 patients require comprehensive pre-transplant evaluation. The decision to perform HSCT on an asymptomatic patients remains controversial due to the unclear natural history of XLP1 in untransplanted individuals. A literature review identified rare cases of untransplanted XLP1 patients who survived into mid-adulthood. Despite surviving typically fatal childhood presentations, these patients remained at risk for late-onset manifestations of XLP1 (108). In another cohort study, the 5-year overall survival (OS) probability from the time of diagnosis was significantly higher in asymptomatic XLP1 patients (100%) compared to symptomatic patients (66.7%) (109). These findings suggest that early HSCT upon diagnosis may offer improved outcomes for asymptomatic XLP1 patients.

### Other potential treatments

#### SLAM Family Inhibitors and insights of controlling EBV infection

Targeted Molecular Interventions: Preclinical studies underscore SLAM family inhibitors as promising therapeutics. Ruffo et al. found that inhibiting DGKα activity in SAP-deficient T cells restores diacylglycerol signaling and alleviates RICD through induction of NR4A1 and NR4A3, thereby identifying DGKα as a potential therapeutic target for reversing the life-threatening EBV-associated immunopathology observed in XLP1 patients (110). Based on this research, Velnati et al. further validated that ritanserin and AMB639752 inhibit DGKa to achieve comparable effects (111). Peled et al. revealed that T cells from XLP1 patients exhibit hyperresponsive to PD-1 signaling, elucidating the critical role of SAP in this process and its underlying mechanism, which suggests that binding partners of the PD-1 cytoplasmic tail may serve as valuable therapeutic targets (51). Neelam et al. showed that RMC-4550 restores T-cells function in XLP1 patient cells and SAP^-/-^ mouse model by enhancing TFH cells function and rescuing cytotoxicity (43).

Hislop et al. demonstrated that inhibiting CD244 facilitates the restoration of CD8^+^ T-cell recognition of EBV-infected target cells, thereby aiding XLP1 patients in controlling EBV infection (112). Additionally, maintaining, restoring, or enhancing CD1d expression on the surface of target cells may improve immune control over EBV-infected cells. This approach could be combined with boosting NKT cell responses via α-galactosylceramide or other agonists (113). More recently, Müller-Durovic et al. found that the Virus-orchestrated nicotinamide adenine dinucleotide (NAD) biosynthesis represents a druggable metabolic vulnerability in EBV-driven B cell transformation, offering potential therapeutic avenues for EBV-related diseases including XLP1 (114). While these approaches can serve as adjuvant treatment, their safe dose, toxicity profiles, and efficacy in humans require further investigation.

#### Gene therapy

Lentiviral vector-mediated SH2D1A gene correction restores SAP expression, thereby rescuing T/NK cell cytotoxicity and humoral immunity in murine and patient-derived cell models, providing novel therapeutic strategies for XLP1 patients (115). Genome editing tools such as CRISPR-Cas9 and CRISPR-Cas12a, combined with AAV6 homology donors, have shown promise in restoring T cells function in XLP1 patients. However, these approaches face challenges, including chromothripsis (extensive chromosome rearrangement restricted to one or a few chromosomes) and dorsal root ganglia toxicity, as well as severe hepatotoxicity associated with high vector doses (116–118). To address these limitations, the use of more efficient AAV vectors that allow for dose sparing or repeated administration of smaller doses, potentially in combination with antibody or B-cell depletion strategies, should be explored.

Organoid-derived T cells with engineered SAP expression demonstrate preclinical efficacy. Recently, a regulated lentiviral vector named XLP-SMART LV, designed to express SAP at therapeutic levels specifically in T, NK, and NKT cells. Transduction of XLP1 patient CD8^+^ T cells or BM CD34^+^ cells with XLP-SMART LVs restored RICD and NK cytotoxicity to wild-type levels, respectively (119, 120). A key advantage of XLP-SMART LVs is their ability to mimic the physiological expression pattern of SAP, ensuring a closer match to the natural context. Nevertheless, the long-term clinical effects of repaired cells on immune function in XLP1 patients remain to be evaluated in clinical trials.

## Research limitations and challenges

Although mutations in the SH2D1A gene have been identified as the cause of XLP1, delineating a definitive relationship between specific genotypes and clinical phenotypes remains challenging. This ambiguity complicates accurate prediction of a patient’s clinical presentation and disease severity through genetic testing. Specifically, different mutations can result in highly variable symptoms among individuals, with some patients experiencing only mild manifestations while others may develop severe complications such as HLH or lymphomas. Therefore, the genotype-phenotype correlation is not a straightforward linear relationship but is influenced by multiple factors, including potential modifier genes and environmental influences.

Current research on XLP1 predominantly relies on small sample sizes, which significantly restricts our understanding of the disease. Specifically, case reports constitute the majority of the literature related to XLP1.These isolated could potentially be aggregated for integrated analysis, such as retrospective cohort studies, to provide a more comprehensive overview of the clinical features, prognosis, and outcomes of XLP1 patients. However, the number of such integrative studies remains extremely limited, thereby hindering the full exploration of the value embedded in reported XLP1 cases. Moreover, special cases involving novel mutations or rare clinical manifestations often lack in-depth mechanistic investigations. A more thorough examination of these cases by relevant researchers is warranted to gather sufficient data for drawing broadly applicable conclusions.

Our understanding of the geographical distribution characteristics of XLP1 patients is insufficient, which may lead to a lack of attention to the disease by medical workers in some high-incidence areas. Future research should focus on larger, multi-center studies to provide more reliable data for the registration and management of XLP1 patients. By the way, these multi-center studies can also explore whether genetic mutations in SH2D1A are related to region and race.

While HSCT is considered an effective treatment for XLP1, its success rate is not fully guaranteed and is influenced by several factors. Despite its potential to cure, the success of HSCT in XLP1 patients is not absolute and can be influenced by various factors, including the timing of the transplant, the presence of active infections, and the patient’s overall health status at the time of the procedure. The decision to proceed with HSCT, especially in asymptomatic patients, remains a complex and debated issue due to the lack of clear natural history data for XLP1 patients who do not undergo transplantation (108). The effectiveness of HSCT can also be impacted by post-transplant complications, such as immune-mediated cytopenias (IMCs), which are a significant cause of morbidity and mortality in pediatric patients undergoing HSCT for both malignant and non-malignant disorders. IMCs are challenging to manage, with many patients showing resistance to first-line treatments like high-dose intravenous steroids, immunoglobulin, and rituximab. The complexity of these complications underscores the need for a deeper understanding of their pathogenesis and the development of tailored therapeutic strategies to improve patient outcomes (121). Furthermore, the success of HSCT in XLP1 patients may be influenced by the underlying genetic mutations affecting immune cell function. For instance, mutations in the SH2D1A gene, resulting in defective NK cell responses against EBV-infected cells. This defect is partly due to the inhibitory function of the 2B4 receptor, which plays a crucial role in NK cell education and immune regulation in XLP1 patients. Understanding these genetic and immunological factors is essential for optimizing HSCT outcomes and developing adjunctive therapies to support immune function in these patients (69). Last but not the least, the establishment of HSCT outcome registries on institutional and national levels may help us obtain a more comprehensive insight into the clinical outcomes of XLP1 patients who undergo HSCT, and provide important data support for the development of clinical trials and cohort studies related to HSCT.

In addition to the traditional treatment methods, the research of targeted therapy has gradually become the focus of attention. However, research in this area is still in its infancy and has not been widely used in clinical practice. Targeted therapy aims to regulate the abnormal response of the immune system by intervening in specific molecular targets, so as to achieve therapeutic purposes. Although preliminary studies suggest this approach has potential applications, more clinical trials are needed to verify its safety and effectiveness. Future research must prioritize multi-center studies to establish genotype-phenotype correlations, validate novel therapies (e.g., gene editing and SLAM inhibitors) in clinical trials, and integrate international registries (e.g., ESID) to enhance global data harmonization.

## Concluding remarks and pepspectives

Current diagnosis and treatment strategies for XLP1 patients focus on eliminating EBV infection, controlling HLH, and awaiting a suitable donor for HSCT. However, the diversity of clinical phenotypes and pathogenesis of XLP1 remains incomplete understood, leading to potential misdiagnosis and missed diagnosis. While existing literature on XLP1 primarily consists of case reports that provide valuable clinical insights, obtaining a comprehensive and integrated understanding of the disease remains challenging. Moreover, XLP1 is associated with SH2D1A gene variants, and our review has identified over 100 mutations, most of which have been confirmed as causative factors for the disease. However, the clinical significance of some mutations remains unclear, and others may yet be undiscovered. Further research into the genetic etiology of XLP1 is likely to uncover new mutation types, potentially enabling better understanding of the disease. Despite the challenges in establishing a definitive correlation between specific genotypes and clinical phenotypes, emerging evidence indicates a potential association, underscoring the need for further multi-center studies. Last but not the least, developing new therapeutic strategies, such as gene-based therapy, is essential for XLP1 patients. Longer-term studies are needed to continued focus on pathogenic mechanisms, genetic associations and clinical features that will advance more precise diagnosis and treatment.
